# Psychiatric disorders and interventions in patients sustaining facial fractures from interpersonal violence

**DOI:** 10.1186/s13005-023-00393-y

**Published:** 2023-10-23

**Authors:** Annamari Arpalahti, Aleksi Haapanen, Kirsi Auro, Anne Abio, Johanna Snäll

**Affiliations:** 1 Department of Oral and Maxillofacial Diseases, University of Helsinki and Helsinki University Hospital, 00029 HUS, Finland; 2 Department of Psychiatry and Adolescent Psychiatry, University of Helsinki and Helsinki University Hospital, Helsinki, Finland; 3 Department of Population Health, National Institute for Health and Welfare, Helsinki, Finland; 4 Injury Epidemiology and Prevention Research Group, Division of Clinical Neurosciences, Turku Brain Injury Centre, Turku University Hospital and University of Turku, Turku, Finland; 5 Heidelberg Institute of Global Health (HIGH), University of Heidelberg, Heidelberg, Germany; 6 Research Centre for Child Psychiatry, University of Turku, Turku, Finland; 7 INVEST Research Flagship Center, University of Turku, Turku, Finland

## Abstract

**Background:**

This retrospective study clarified patients´ psychiatric morbidity in IPV-related facial fractures; in particular, their additional psychiatric care. We hypothesized that patients in need of additional support can be identified, allowing overall care processes to be improved.

**Methods:**

Patients’ age, sex, anamnestic psychiatric disorders, history of substance abuse, and psychiatric interventions were recorded, as well as the perpetrator, location, time of day, assault mechanism, fracture type, treatment, and associated injuries.

**Results:**

In all, 807 adult patients were included in the study. Of these, 205 patients (25.4%) had anamnestic psychiatric disorders that were associated independently with female sex (OR 1.95, 95% CI 1.12, 3.41; *p* = 0.019) or history of substance abuse (OR 5.82, 95% CI 4.01, 8.46; *p* < 0.001). Patients with anamnestic psychiatric disorder were more likely to be subjected to severe violence, with an increased risk for combination fractures (OR 2.51, 95% CI 1.30, 4.83; *p* = 0.006). Of all patients, 61 (7.6%) received a psychiatric intervention within the first 12 months. The most common reasons for intervention were anxiety/fear and psychotic symptoms, surfacing within one month in 57% of patients. Anamnestic psychiatric disorders (OR 2.00, 95% CI 1.04, 3.82; *p* = 0.036), severe mental illnesses (OR 2.45, 95% CI 1.04, 5.77; *p* = 0.040), and use of an offensive weapon (OR 2.11, 95% CI 1.11, 4.02; *p* = 0.023) were the strongest independent predictors of psychiatric intervention.

**Conclusions:**

Our results emphasize the need for more structured treatment protocols for patients sustaining IPV injury. Special attention is recommended for patients with anamnestic psychiatric disorders, severe mental illnesses, and those assaulted with an offensive weapon.

## Background

Interpersonal violence (IPV) has long maintained its rank as a notable etiology of facial fractures, the sequelae of which have gained attention from both the scientific community and mainstream media. IPV, as well as other traumatic events, can trigger such psychiatric symptoms as anxiety, posttraumatic stress disorder (PTSD) [1, 2], paranoia [3], suicidal ideation, and depression [4].

Facial injury and their psychiatric aftermath [2, 5] – the psychiatric toll of IPV in particular [6, 7] – have been investigated extensively. Estimates of their prevalence vary; for example, for PTSD in America the estimates range from 3.6% for one year to 7.8% over the lifetime [8]. The corresponding figures for Italy are 0.7% and 2.4% [9]. For Europe, the one-year estimate is 1.1% [10]. Acute psychiatric symptoms [11] and female sex [11, 12] have been suggested to raise the probability of PTSD in trauma cases with disfiguring facial injuries [12]. It should be noted, however, that these studies often exclude patients with certain pre-existing psychiatric diagnoses and substance abuse, patient groups potentially with increased need for additional care resources. Thus, recognizing victims of IPV at increased risk and allowing them better access to suitable services should be important objectives in healthcare [13].

Characteristics of the perpetrator and the victim can influence the event. The general behavior – often subconscious – of the victim can affect the types of situations they encounter [14]. More specific characteristics, such as severe mental illness, can also play a role, increasing the odds of becoming a victim of violent acts [15]. Regarding schizophrenia and psychosis patients involved in violent events, over one-third of these events occur when the patients are in their first-ever episode [16] – a factor to consider in both perpetrators and victims. A recent study also found that psychiatric history of the victim coincided with more severe injuries and longer stay in hospital [17]. Additionally, factors affecting at the moment of trauma or immediately after have been suggested to have an even stronger effect on the development of psychiatric symptoms after a traumatic event [18].

The purpose of this study was to clarify psychiatric morbidity in IPV-related facial fracture patients. In particular, we clarified patients´ additional psychiatric care in the form of a psychiatric intervention after facial fracture. We hypothesized that patients needing additional support can be identified based on background variables, allowing us to improve overall care processes.

## Methods

### Study design

A retrospective study of assaulted facial fracture patients was designed and implemented. Patient data of all facial fracture patients evaluated in a tertiary trauma center in 2013–2018 were reviewed.

### Inclusion and exclusion criteria

Patients aged 18 years or more who sustained facial fractures from IPV were included in the study. Both clinical examination and radiological imaging were required for fracture diagnosis.

### Study variables

The main outcome variable was psychiatric intervention. This was in the form of a psychiatric evaluation by a psychiatrist or psychiatric nurse during fracture care or after an acute exacerbation of the patient’s mental health occurring within 12 months of the facial fracture.

The primary predictor variable was the preceding psychiatric morbidity defined as an anamnestic disease or disorder requiring psychiatric treatment. These were categorized as follows: psychiatric illnesses included psychotic disorders, mood disorders, anxiety disorders, personality disorders and hyperkinetic disorders. Psychiatric morbidities with psychotic components were classified as severe mental illnesses (SMI): these included psychotic disorders, bipolar disorder, and severe depression with a psychotic component. Substance abuse or alcohol delirium was not classified as a psychiatric morbidity unless it required psychiatric treatment.

Additional predictor variables were history of substance and/or alcohol abuse (yes/no) and the following injury-related variables: perpetrator(s) (known by patient; stranger; not specified), location of assault (home or apartment; bar or indoor public space; outdoors; not specified), time of day (daytime 6 am to 6 pm; evening 6 pm to 10 pm; night 10 pm to 6 am; not specified), assault mechanism (single hit; multiple hits or different mechanisms; not specified) and use of an offensive weapon (yes/no).

Explanatory variables were age, sex, fracture type (mandibular; midfacial; upper third; combination of facial thirds), surgical treatment for facial fracture (yes/no), and associated injury (yes/no). Associated injuries were defined as brain injuries and injuries occurring elsewhere in the body.

In addition, specific reasons for psychiatric interventions and their timeline were reported. The reasons included psychiatric morbidity, occurrence of a new psychiatric diagnosis, or worsened psychiatric disorder.

### Statistical analysis

Descriptive statistics were estimated with percentages for categorical variables, and median and interquartile range for the continuous variable (age), which was not normally distributed. Pearson Chi-square tests were used in cross-tabulations between the psychiatric intervention variable and the predictor and explanatory variables. However, Fisher’s Exact was used when a cell had five or less observations. Binary logistic regression was used to estimate the association between the predictor and additional or explanatory variables, as well as association between the outcome and predictor or explanatory variables. Variables in the final multivariable model were determined based on *p* ≤ 0.1. The Homer-Lemeshow test, applied to determine the goodness fit of the model, revealed a good fit. Furthermore, the Variance Inflation Factor (VIF) was used to test for multicollinearity in the final model. All the predictor/explanatory variables were found to have VIF value of less than 7, suggesting collinearity was not a serious problem. The analysis was conducted using Stata 17 (StataCorp, TX, USA).

## Results

Altogether 807 patients fulfilled the inclusion criteria and were included in the study (see Table 1); 722 (89.5%) were men, and patients’ mean age was 34.86 years. Patients were most likely assaulted by a stranger (51.9%), outdoors (31.2%), at night (58.5%), and by multiple hits (57.5%). They had fractures most frequently in the middle third of the face (56.8%) and were treated surgically (52.7%).

**Table 1:** General characteristics of the patient population with regard to anamnestic psychiatric disorders

	**All patients**	**Patients with anamnestic psychiatric disorders**			**Patients without anamnestic psychiatric disorders**			***P*-value**
		***n***	**%**	**% of n**	***n***	**%**	**% of n**	
**Patient-related**	**807**	**205**		**25.4**	**602**		**74.6**	
**Age**								0.454
Average	34.86 (SD = 12.08)	35.14 (SD = 12.3)			34.76 (SD = 12.0)			
Median	32.23	33.12			32.09			
Interquartile range (IQR)		25.7; 42.2			25.0; 43.2			
Range	18.0; 87.4	18.0; 87.4			18.1; 77.7			
**Sex**								**0.003**
Male	722 (89.5)	172	83.9	23.8	550	91.4	76.2	
Female	85 (10.5)	33	16.1	38.8	52	8.6	61.2	
**History of substance abuse**								** < 0.001**
Yes (alcohol and/or substance abuse)	252 (31.2)	123	60.0	48.8	129	21.4	51.2	
No	555 (68.8)	82	40.0	14.8	473	78.6	85.2	
**Assault-related**								
**Perpetrator**								0.082
Known to the patient	216 (26.8)	66	32.2	30.6	150	24.9	69.4	
Stranger	419 (51.9)	103	50.2	24.6	316	52.5	75.4	
Not specified	172 (21.3)	36	17.6	20.9	136	22.6	79.1	
**Location**								0.200
Home/apartment	119 (14.7)	38	18.5	31.9	81	13.5	68.1	
Bar/indoor public space	247 (30.6)	54	26.3	21.9	193	32.1	78.1	
Street/Outdoors	252 (31.2)	62	30.2	24.6	190	31.6	75.4	
Not specified	189 (23.4)	51	24.9	27.0	138	22.9	73.0	
**Time of day**								0.173
Morning/Daytime (6–18)	94 (11.6)	25	12.2	26.6	69	11.5	73.4	
Evening (18–22)	133 (16.5)	40	19.5	30.1	93	15.4	69.9	
Night (22–6)	472 (58.5)	107	52.2	22.7	365	60.6	77.3	
Not specified	108 (13.4)	33	16.1	30.6	75	12.5	69.4	
**Assault mechanism**								0.319
Single hit	250 (31.0)	56	27.3	22.4	194	32.2	77.6	
Multiple hits/mechanism	464 (57.5)	127	62.0	27.3	337	56.0	72.6	
Not specified	93 (11.5)	22	10.7	23.7	71	11.8	76.3	
**Offensive weapon**	116 (14.4)	41	20.0	35.3	75	12.5	64.7	**0.008**
No	691 (85.6)	164	80.0	23.7	527	87.5	76.3	
**Fracture types**								
Lower third	273 (33.8)	52	25.4	19.0	221	36.7	81.0	0.007
Middle third	458 (56.8)	129	62.9	28.2	329	54.7	71.8	
Upper third	14 (1.7)	2	1.0	14.3	12	2.0	85.7	
Combination	62 (7.7)	22	10.7	35.5	40	6.6	64.5	
**Treatment**								0.024
Surgical	425 (52.7)	94	45.9	22.1	331	55.0	77.9	
Non-surgical	382 (47.3)	111	54.1	29.1	271	45.0	70.9	
**Associated injury**								0.049
Yes	123 (15.2)	40	19.5	32.5	83	13.8	67.5	
No	684 (84.8)	165	80.5	24.1	519	86.2	75.9	

Patient population *n* = 807, % is the column percentage (i.e., the percentage of patients within each group with the given characteristic); '% of n' is the row percentage (i.e., the distribution of patients with the given characteristic between the two groups)

### Anamnestic psychiatric diagnoses

In all, 205 patients (25.4%) had anamnestic psychiatric disorders (see Table 1). Compared with patients without anamnestic psychiatric diagnoses, the most distinguishing features were sex (*p* = 0.003), history of substance abuse (*p* < 0.001), use of an offensive weapon (*p* = 0.008), fracture type (*p* = 0.007), and associated injuries (*p* = 0.049).

The strongest indicators of anamnestic psychiatric disorders in the unadjusted model were history of substance abuse (Unadjusted Odds Ratio 5.50, 95% Confidence Interval 3.91, 7.73; *p* < 0.001), combination fractures of the facial thirds (OR 2.34, 95% CI 1.28, 4.27; *p* = 0.006), and female sex (OR 2.03, 95% CI 1.27, 3.24; *p* = 0.003). Use of an offensive weapon (OR 1.76, 95% CI 1.16, 2.67; *p* = 0.008) and middle-third fractures (OR 1.67, 95% CI 1.16, 2.40; *p* = 0.006) were also more likely in patients with pre-existing psychiatric disorders. In the anamnestic psychiatric disorder group, it was also less likely for the relationship between the victim and perpetrator not to be specified (OR 0.60, 95% CI 0.38, 0.96; *p* = 0.033), for the assault to take place in a bar (OR 0.60, 95% CI 0.37, 0.97; *p* = 0.038), and for the fracture to be treated surgically (OR 0.69, 95% CI 0.50, 0.95; *p* = 0.024).

Congruently, in multivariate analysis, female sex (Adjusted OR 1.95, 95% CI 1.12, 3.41; *p* = 0.019), history of substance abuse (OR 5.82, 95% CI 4.01, 8.46; *p* < 0.001), and fractures with combination of facial thirds (OR 2.51, 95% CI 1.30, 4.83; *p* = 0.006) were statistically significant. The univariate and multivariate analyses in these patients can be seen in Table 2.

**Table 2:** Results of univariate and multivariate analyses of study variables with regard to anamnestic psychiatric disorders

Variable	**Unadjusted OR**	**95% CI**	***P***-value	**Adjusted OR**	**95% CI**	***P*-value**
Age	1	0.99; 1.02	0.693	//	//	//
Sex (Ref: men)	2.03	1.27; 3.24	0.003	1.95	1.12; 3.41	**0.019**
History of substance abuse	5.50	3.91; 7.73	< 0.001	5.82	4.01; 8.46	** < 0.001**
**Assault-related**						
**Perpetrator**						
Known to patient	1			1		
Stranger	0.74	0.51; 1.07	0.107	0.97	0.61; 1.52	0.883
Not specified	0.60	0.38; 0.96	0.033	0.73	0.42; 1.25	0.253
**Location**						
Home/apartment	1			1		
Bar/indoor public space	0.60	0.37; 0.97	0.038	1.14	0.62; 2.09	0.681
Street/outdoors	0.70	0.43; 1.12	0.139	0.87	0.47; 1.60	0.655
Not specified	0.79	0.48; 1.30	0.351	0.91	0.50; 1.66	0.751
**Time of day**						
Morning/daytime (6am-6 pm)	1					
Evening (6 pm-10 pm)	1.19	0.66; 2.13	0.568	//	//	//
Night (10 pm-6am)	0.81	0.49; 1.34	0.412	//	//	//
Not specified	1.21	0.66; 2.24	0.535	//	//	//
**Assault mechanism**						
Single hit	1					
Multiple hits/mechanism	1.31	0.91; 1.87	0.147	//	//	//
Not specified	1.07	0.61; 1.89	0.805	//	//	//
Offensive weapon	1.76	1.16; 2.67	0.008	1.04	0.63; 1.70	0.885
**Fracture type**						
Lower third	1			1		
Middle third	1.67	1.16; 2.40	0.006	1.44	0.97; 2.15	0.072
Upper third	0.71	0.15; 3.26	0.658	0.54	0.10; 2.76	0.458
Combination of facial thirds	2.34	1.28; 4.27	0.006	2.51	1.30; 4.83	**0.006**
Treatment (Ref: non-surgical)	0.69	0.50; 0.95	0.024	//	//	//
Associated injuries	1.52	1.00; 2.30	0.050	1.14	0.71; 1.83	0.575

*OR* Odds ratio, *CI* Confidence interval

**Fig. 1:**
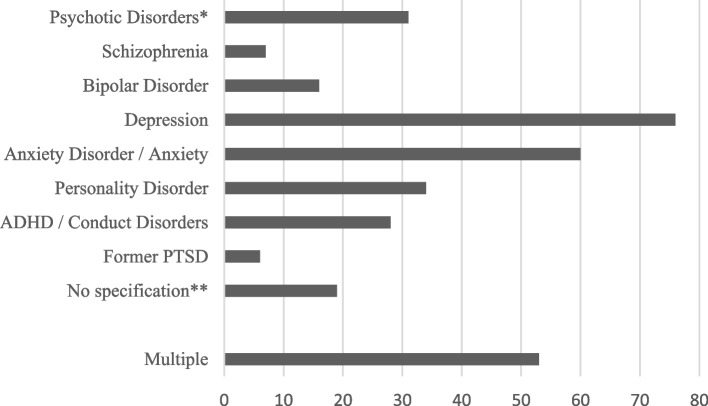
Specific diagnoses of 205 patients with anamnestic psychiatric disorders. Abbreviation: PTSD = post-traumatic stress disorder. * excluding bipolar disorder or depression with a psychotic component; ** psychiatric disorder inferred but not specified

In all, 53 of 205 patients with psychiatric history (25.9%) had multiple anamnestic psychiatric disorders, most commonly depression, anxiety, and personality disorders (Fig. 1).

### Psychiatric intervention

Table 3 presents data comparing patients who received a psychiatric intervention and patients who did not. Of all patients, 61 (7.6%) received a psychiatric intervention, with a mean age of 38.2 (IQR 27.0 – 46.3). Compared with patients who did not receive a psychiatric intervention, the groups differed in terms of age (*p* = 0.011), history of substance abuse (*p* < 0.001), assault mechanism (*p* = 0.037), whether an offensive weapon was used (*p* < 0.001), and associated injuries (*p* = 0.035).

**Table 3:** General characteristics of the patient population with regard to psychiatric intervention

	**Patients who received psychiatric intervention**			**Patients without psychiatric intervention**			***P*-value**
	***n***	**%**	**% of n**	***n***	**%**	**% of n**	
**Patient-related**	61		7.6	746		92.4	
**Age**							**0.011**
Average	38.2 (SD = 11.0)			34.6 (SD = 12.1)			
Median	37.5			32.0			
Interquartile range (IQR)	27.0; 46.3			24.7; 42.2			
Range	20.1; 62.2			18.0; 87.4			
**Sex**							0.264
Male	52	85.2	6.4	670	89.8	83.0	
Female	9	14.8	1.1	76	10.2	9.4	
**Psychiatric history (yes)**	29	47.5	3.6	176	23.6	21.8	** < 0.001**
Severe (SMI)	12	19.7	1.5	39	5.2	4.8	** < 0.001**
No	32	52.5	4.0	570	76.4	70.6	
**History of substance abuse**							**0.001**
Yes (alcohol and/or substance abuse)	31	50.8	3.8	221	29.6	27.4	
No	30	49.2	3.7	525	70.4	65.1	
**Assault-related**							
**Perpetrator**							0.805
Known to the patient	16	26.2	2.0	200	26.8	24.8	
Stranger	30	49.2	3.7	389	52.1	48.2	
Not specified	15	24.6	1.9	157	21.0	19.5	
**Location**							0.324
Home/apartment	12	19.7	1.5	107	14.3	13.3	
Bar/indoor public space	16	26.2	2.0	231	31.0	28.6	
Street/Outdoors	15	24.6	1.9	237	31.8	29.4	
Not specified	18	29.5	2.2	171	22.9	21.2	
**Time of day**							0.099
Morning/Daytime (6–18)	13	21.3	1.6	81	10.9	10.0	
Evening (18–22)	10	16.4	1.2	123	16.5	15.2	
Night (22–6)	30	49.2	3.7	442	59.2	54.8	
Not specified	8	13.1	1.0	100	13.4	12.4	
**Assault mechanism**							0.037
Single hit	10	16.4	1.2	240	32.2	29.7	
Multiple hits/mechanism	42	68.9	5.2	422	56.6	52.3	
Not specified	9	14.8	1.1	84	11.3	10.4	
**Offensive weapon**	18	29.5	2.2	98	13.1	12.1	** < 0.001**
No	43	70.5	5.3	648	86.9	80.3	
**Fracture types**							0.348
Lower third	15	24.6	1.9	258	34.6	32.0	
Middle third	41	67.2	5.1	417	55.9	51.7	
Upper third	1	1.6	0.1	13	1.7	1.6	
Combination of facial thirds	4	6.6	0.5	58	7.8	7.2	
**Treatment**							0.617
Surgical	34	55.7	4.2	391	52.4	48.5	
Non-surgical	27	44.3	3.3	355	47.6	44.0	
**Associated injury**							0.035
Yes	15	24.6	1.9	108	14.5	13.4	
No	46	75.4	5.7	639	85.7	79.2	

patient population *n* = 807, % is the column percentage (i.e., the percentage of patients within each group with the given characteristic; '% of n' is the row percentage (i.e., the distribution of patients with the given characteristic between the two groups)

In the unadjusted model, presented in Table 4, patients with SMIs were four times more likely to receive a psychiatric intervention (OR 4.44; 95% CI 2.19, 9.02; *p* < 0.001). Patients with anamnestic psychiatric disorders were nearly three times more likely to receive a psychiatric intervention (OR 2.94, 95% CI 1.73, 4.99; *p* < 0.001) and two times more likely if they had a history of substance abuse (OR 2.45, 95% CI 1.45, 4.15; *p* = 0.001). Patients who were hit multiple times, as opposed to a single hit, were twice as likely to receive an intervention (OR 2.39, 95% CI 1.18, 4.85; *p* = 0.016). Patients assaulted at night between 10 pm and 6 am were less likely to receive a psychiatric intervention (OR 0.42, 95% CI 0.21, 0.84; *p* = 0.015).

**Table 4:** Results of univariate and multivariate analyses of study variables with regard to psychiatric intervention

**Variable**	**Unadjusted OR**	**95% CI**	***P*-value**	**Adjusted OR**	**95% CI**	***P*-value**
Age	1.02	1.00; 1.04	0.025	1.02	1.00; 1.04	0.130
Sex (Ref: men)	1.53	0.72; 3.22	0.267	//	//	//
Anamnestic psychiatric disorders	2.94	1.73; 4.99	< 0.001	2.00	1.04; 3.82	**0.036**
Severe mental illness	4.44	2.19; 9.02	< 0.001	2.45	1.04; 5.77	**0.040**
History of substance abuse	2.45	1.45; 4.15	0.001	1.34	0.72; 2.49	0.353
**Assault-related**						
**Perpetrator**						
Known to patient	1					
Stranger	0.96	0.51; 1.81	0.909	//	//	//
Not specified	1.19	0.57; 2.49	0.636	//	//	//
**Location**						
Home/apartment	1					
Bar/indoor public space	0.62	0.28; 1.35	0.228	//	//	//
Street/outdoors	0.56	0.26; 1.25	0.157	//	//	//
Not specified	0.94	0.43; 2.03	0.872	//	//	//
**Time of day**						
Morning/daytime (6am-6 pm)	1			1		
Evening (6 pm-10 pm)	0.51	0.21; 1.21	0.126	0.40	0.16; 0.998	0.050
Night (10 pm-6am)	0.42	0.21; 0.84	0.015	0.50	0.24; 1.05	0.068
Not specified	0.50	0.20; 1.26	0.142	0.50	0.19; 1.32	0.161
**Assault mechanism**						
Single hit	1			1		
Multiple hits/mechanism	2.39	1.18; 4.85	0.016	1.81	0.86; 3.79	0.118
Not specified	2.57	1.01; 6.54	0.048	1.87	0.70; 5.01	0.215
Offensive weapon	2.77	1.53; 4.99	0.001	2.11	1.11; 4.02	**0.023**
**Fracture type**						
Lower third	1					
Middle third	1.69	0.92; 3.12	0.092	//	//	//
Upper third	1.32	0.16; 10.80	0.794	//	//	//
Combination of facial thirds	1.19	0.38; 3.71	0.769	//	//	//
Treatment (Ref: non-surgical)	1.14	0.68; 1.93	0.617	1.70	0.96; 3.02	0.069
Associated injuries	1.93	1.04; 3.57	0.037	1.46	0.74; 2.86	0.274

*OR* Odds ratio, *CI* Confidence interval

Congruently, in the multivariate analysis, if the patient had an anamnestic psychiatric disorder (OR 2.00; 95% CI 1.04, 3.82; *p* = 0.036) or an SMI (OR 2.45; 95% CI 1.04, 5.77; *p* = 0.040) or if an offensive weapon was used (OR 2.11, 95% CI 1.11, 4.02; *p* = 0.023), they were twice as likely to receive a psychiatric intervention.

**Fig. 2:**
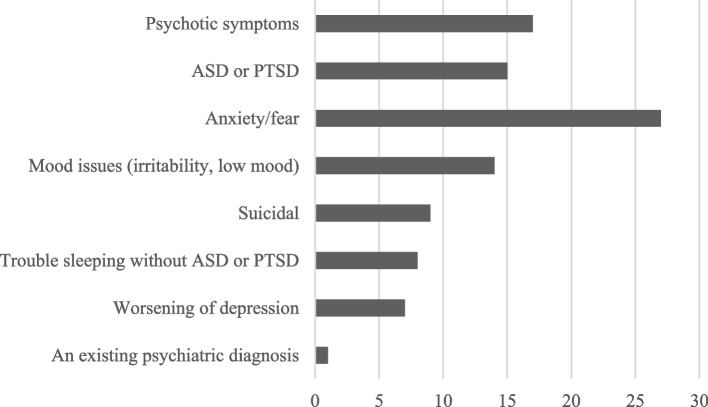
Reasons and symptoms underlying psychiatric intervention in 61 patients with facial fracture. Abbreviations: ASD = acute stress disorder; PTSD = post-traumatic stress disorder

**Fig. 3:**
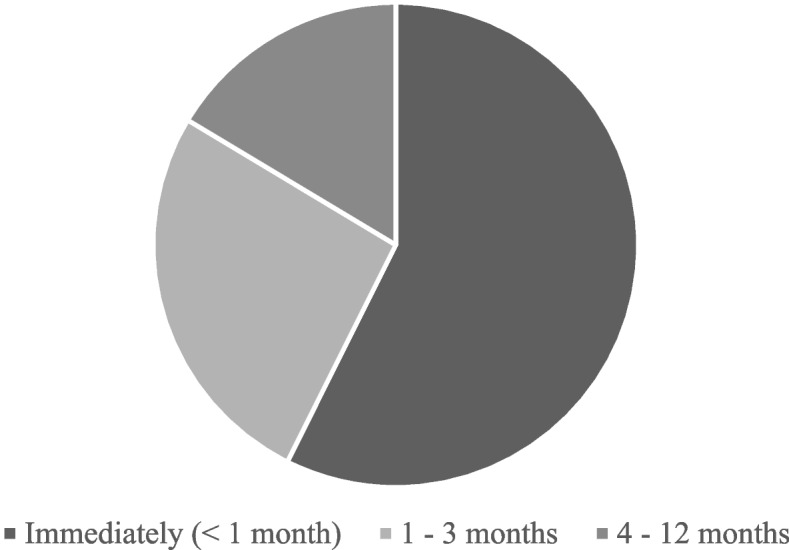
Chart of onset of psychiatric symptoms in patients who received an intervention

Symptoms and underlying reasons for psychiatric interventions are presented in Fig. 2. The most common reasons included feelings of fear, anxiety, psychotic symptoms, and traumatic stress responses, in the form of either acute stress disorder (ASD) or PTSD. Psychotic symptoms included hallucinations and delusions that were not clearly part of traumatic brain injury symptoms. As shown in Fig. 3, the onset of psychiatric symptoms was within one month of assault in over half (57.4%) of the cases.

## Discussion

This study aimed to clarify psychiatric morbidity in IPV-related facial fracture patients. In particular, we clarified patients´ additional psychiatric care in the form of psychiatric intervention after facial fracture. We hypothesized that patients needing additional support can be identified based on background variables, allowing overall care processes to be improved.

Our results revealed that female patients with IPV-related facial fracture are twice as likely as males to have anamnestic psychiatric disorders. Patients with preceding psychiatric history sustain more severe assaults with combination fractures of the facial thirds, and they are more likely to have a history of substance abuse than other IPV-related facial fracture patients. Both anamnestic psychiatric disorders and SMIs as well as the use of an offensive weapon increased the probability that patients would receive psychiatric intervention.

### Assault and later psychiatric symptoms

The after-effects on the psyche after traumatic events typically include anxiety, PTSD [1, 2], paranoia [3], suicidal ideation, and depression [4] as well as a multitude of mental symptoms such as distress, dissociation, and mental defeat [13]. However, a lack of research on these predisposing factors to various psychiatric symptoms after traumatic events, excluding PTSD, has been noted [19]. History of mental illness has been established to be the most significant predisposing factor for severe psychiatric symptoms following traumatic events (defined as three or more psychiatric diagnoses after the traumatic event) [19]. The risk for psychiatric symptoms is increased especially if there is a preceding personality disorder, major depressive disorder, or antidepressant medication [19]. Supporting this, we found that patients with anamnestic psychiatric disorders, particularly SMIs, often required psychiatric intervention after experiencing IPV.

Especially after assault, psychiatric disorders include paranoia and PTSD [3]. Usually, the follow-up period in studies is limited to a couple of months or years, but the after-effects can disperse and evolve over a longer time-span; an American study on the lifetime effect of assault found that depression, anxiety disorders, and substance abuse disorders were more common in patients with a history of assault than in patients without [20]. In our data, in addition to traumatic stress reactions (ASD or PTSD), psychotic symptoms (delusions, hallucinations, extensive paranoia) were among the most prevalent psychiatric manifestations post-assault. The most common individual symptoms included anxiety and fear (44.3%), mood issues (30.0%), and problems with sleep (13.1%) (Fig. 2**)**. The majority of interventions were carried out within one month – however, nearly half of the patients developed psychiatric symptoms at 1–12 months, emphasizing the value of consistent follow-ups (Fig. 3). The relatively high incidence of psychoses may stem partly from our inclusion of patients with both history of substance abuse and all types of psychiatric disorders, including psychotic conditions. Due to the difficulty of these patients in committing to follow-ups and their potentially erratic behavior, many studies may exclude them, especially from prospective studies. Despite this, it should be noted that some of the psychoses developed without a pre-existing psychotic disorder or history of substance abuse.

### Risk factors for violence in patients with psychiatric morbidities

In patients with behavioral health disorders, including psychiatric disorders, assaults have been found to cause facial fractures in over one-fourth of the patients, significantly more often than in the control group [21]. Moreover, SMIs have been suggested to raise the risk for being a victim of a crime in general [15], and death by homicide is nearly three times more frequent in people with psychiatric disorders than in the general population [22]. These types of studies have also included psychiatric patients and prison inmates and have compared people with and without mental illnesses [6].

A recent article reported a connection between psychiatric morbidity and the severity of injuries from trauma [17]. In our data, one-quarter of patients had an anamnestic psychiatric disorder, 24.8% of which were SMIs. The link to increased severity of injuries can also be seen in our data; the risk for combination fractures was elevated in patients with anamnestic psychiatric disorders. A similar trend was observed in patients who received psychiatric intervention; they were more often assaulted with an offensive weapon and had associated injuries. They were also less likely to be assaulted with a single hit and had more mid-face fractures, although the last finding did not reach statistical significance.

Some psychiatric disorders have been associated with a tendency for more aggressive behavior than others. Regarding psychoses, it has been suggested that roughly a third of psychotic patients involved in assaults are experiencing psychosis for the first time [16]. A threat of violence can also be present in certain personality disorders such as pathological narcissism, psychopathy, borderline personality disorder, and paranoid/schizotypal personality disorder [23]. For example, among prison inmates, over 70% had an antisocial personality disorder and they carried out more acts of aggression than the rest of the inmates [24]. Poor impulse control [25] and poor metacognitive functions [26] have also been linked to violent tendencies. Psychiatric comorbidities, like schizophrenia and substance abuse disorders (SUDs), can also increase the risk for violence [27]. The prevalence of personality disorders in assaulted facial fracture patients in our data (4.6% of all patients, see Fig. 1) is dramatically different from the figure for antisocial disorder among prison inmates (70%) [24]. This is likely due to screening bias – prison inmates are screened for different psychiatric disorders, whereas the psychiatric history of patients in our study was not screened systematically.

History of substance abuse increased the probability of both having anamnestic psychiatric conditions and receiving a psychiatric intervention, although the latter reached statistical significance only in the univariate analysis. Previous studies support these findings; IPV has been suggested to be the most common etiology in SUD patients with maxillofacial injuries [28], and SUDs have also been shown to be a significant risk factor for facial fractures, slightly more than male sex [28]. In our data, nearly one-third of patients (31.2%) had a history of substance abuse, and such a large proportion should warrant more convenient clinical pathways to substance abuse psychiatry. However, it is also important to note that a large proportion (49.2%) of the patients who received a psychiatric intervention did not have an SUD.

### Guiding patients to mental health services and the impact of treatment

A recent study showed that facial fracture patients with psychiatric disorders had more serious injuries, requiring more extensive treatment [17]. Another study stated that patients with behavioral health disorders, including psychiatric disorders, were three times more likely to be transferred to a psychiatric facility after a facial fracture than those in the control group without behavioral health disorders [21]. Apart from pre-trauma factors, factors related directly to the moment of trauma and its immediate aftermath have been suggested to have an even greater impact [18]. By using Hospital Anxiety and Depression Scale scores, it has also been proposed that facial trauma patients are at a greater risk of developing depression than elective surgery patients [29]. Taken together, more clearly targeted clinical pathways are indicated for patients with anamnestic psychiatric disorders.

### Limitations of the study

The retrospective study design limits detailed conclusions, and the prevalence of psychiatric illness may be underestimated. Firstly, patients who received psychiatric care outside the University Hospital were not included. With this, we aimed at evaluating specifically the effort of specialized health care. Secondly, since the study was retrospective, only the most prevalent cases, meaning those that had symptoms so prominent that they were detected and described in the patient files, were included. A survey on these symptoms during the treatment period could have revealed some more minor symptoms or possibly symptoms that were not outwardly evident. After a violent event, about one-third of people who are exposed to nonsexual violence seek the help of a mental health professional [13]. This contrast in quantity between seeking help and our results on psychiatric evaluation is large but evidently due to the difference in study design (prospective vs. retrospective set-up). Thus, the incidence of psychiatric symptoms after IPV in our study is likely to be higher than that reported. Thirdly, a direct comparison of our results with earlier studies is challenging due to differences in the definitions of psychiatric symptoms and diseases ( 13). Prospective studies could help elucidate the need for additional treatment in the future. Additionally, the authors would like to emphasize that while the method of how additional psychiatric care is provided is relevant, it falls outside the scope of this study, which is to focus on the types of facial fracture patients that required additional care the most.

## Conclusions

One-fourth (25.4%) of assaulted facial fracture patients in our data had anamnestic psychiatric disorders. Their assaults on average were more severe, resulting in more combination fractures. Psychiatric intervention was received by 7.6% of all assaulted facial fracture patients. Although the intervention was more likely when an anamnestic psychiatric disorder was present, a considerable proportion (52.5%) of patients who received a psychiatric intervention did not have pre-existing disorders. The most common psychiatric symptoms after the assault were psychotic and traumatic stress symptoms. Close evaluation is recommended, especially in patients with anamnestic psychiatric disorders or SMIs or those assaulted with an offensive weapon. We encourage healthcare personnel treating facial fractures to construct targeted protocols with psychiatric services to evaluate psychiatric health in all IPV victims.

## Data Availability

All data, excluding a chart containing individualizing patient information, generated or analysed during this study are included in this published article (and its supplementary information files).

## Abbreviations

IPV: Interpersonal violence
PTSD: Posttraumatic stress disorder
SMI: Severe mental illness
VIF: Variance inflation factor
OR: Odds ratio
ASD: Acute stress disorder
SUD: Substance abuse disorder
